# Energy Metabolism and Hyperactivation of Spermatozoa from Three Mouse Species under Capacitating Conditions

**DOI:** 10.3390/cells11020220

**Published:** 2022-01-10

**Authors:** Ester Sansegundo, Maximiliano Tourmente, Eduardo R. S. Roldan

**Affiliations:** 1 Department of Biodiversity and Evolutionary Biology, Museo Nacional de Ciencias Naturales, Spanish Research Council (CSIC), 28006 Madrid, Spain; estersansegundohernando@gmail.com; 2 Centro de Biología Celular y Molecular, Facultad de Ciencias Exactas, Físicas y Naturales, Universidad Nacional de Córdoba, Cordoba X5016GCA, Argentina; 3 Instituto de Investigaciones Biológicas y Tecnológicas (IIByT), Consejo Nacional de Investigaciones Científica y Técnicas (CONICET), Cordoba X5016GCA, Argentina

**Keywords:** capacitation, hyperactivation, bioenergetics, ATP, sperm swimming

## Abstract

Mammalian sperm differ widely in sperm morphology, and several explanations have been presented to account for this diversity. Less is known about variation in sperm physiology and cellular processes that can give sperm cells an advantage when competing to fertilize oocytes. Capacitation of spermatozoa, a process essential for mammalian fertilization, correlates with changes in motility that result in a characteristic swimming pattern known as hyperactivation. Previous studies revealed that sperm motility and velocity depend on the amount of ATP available and, therefore, changes in sperm movement occurring during capacitation and hyperactivation may involve changes in sperm bioenergetics. Here, we examine differences in ATP levels of sperm from three mouse species (genus *Mus*), differing in sperm competition levels, incubated under non-capacitating and capacitating conditions, to analyse relationships between energetics, capacitation, and swimming patterns. We found that, in general terms, the amount of sperm ATP decreased more rapidly under capacitating conditions. This descent was related to the development of a hyperactivated pattern of movement in two species (*M. musculus* and *M. spicilegus*) but not in the other (*M. spretus*), suggesting that, in the latter, temporal dynamics and energetic demands of capacitation and hyperactivation may be decoupled or that the hyperactivation pattern differs. The decrease in ATP levels during capacitation was steeper in species with higher levels of sperm competition than in those with lower levels. Our results suggest that, during capacitation, sperm consume more ATP than under non-capacitating conditions. This higher ATP consumption may be linked to higher velocity and lateral head displacement, which are associated with hyperactivated motility.

## 1. Introduction

Competition between spermatozoa to fertilize oocytes [1,2,3,4,5] may influence the evolution of sperm structure [6,7] and performance [7,8,9]. Sperm competition may select for faster swimming sperm, which may be the result of changes in various components of sperm morphology [10,11,12,13,14] or bioenergetics [15]. Despite some characterization of sperm performance upon release from the male reproductive tract (i.e., from the epididymis or after ejaculation), in a comparative and evolutionary context, there has been little advance in evolutionary studies examining post-ejaculatory stages in the life of the sperm cell.

Mammalian sperm are not able to engage in fertilization immediately after ejaculation since fertilizing ability of sperm cells is acquired during a period of residence in the female reproductive tract [16]. These physiological changes may be induced by uterine and/or oviductal cells, fluids, and the female gamete itself [16] and are collectively referred to as “capacitation” [17,18,19,20]. Historically, capacitation was defined as the period of time that spermatozoa require to undergo these morphological and physiological changes in vivo or in vitro [21]. This definition has suffered transformations throughout the years to include or discriminate other physiological milestones in the sperm lifetime, such as the acrosome reaction and changes in sperm swimming patterns [22,23,24]. The process of capacitation correlates with changes in the sperm intracellular environment, i.e., ion concentrations [25,26], increased membrane fluidity [27,28,29], sperm metabolism [30,31], and motility [22].

Sperm capacitation can be achieved in vitro by incubating spermatozoa in a culture medium that mimics the composition of the fluids in the female reproductive tract [24,32]. In vitro capacitation of mouse sperm requires a medium with a glycolysable metabolic substrate, usually glucose [30,33], a salt solution, physiologically active ions, such as Ca^2+^ and HCO_3_^−^, and a protein source [22,27,34,35,36]. In addition, bovine serum albumin (BSA) is needed to induce changes in the levels of cholesterol of the plasma membrane, causing changes in its fluidity and increasing permeability to Ca^2+^ and HCO_3_^−^. The resulting increase in ionic levels within the intracellular compartment stimulates the activity of adenylyl cyclase, increasing cAMP, protein phosphorylation kinase A, and protein tyrosine phosphorylation [35,36,37,38].

Spermatozoa stored in the cauda epididymis before ejaculation are immotile and they express motility after they are exposed to seminal plasma, or a chemically equivalent medium [39]. This type of movement, termed “activated” motility, consists of vigorous and symmetric flagellar movements, which result in a nearly linear trajectory [24,40,41]. A characteristic feature of capacitation is the acquisition of a different pattern of sperm motility, known as “hyperactivation” [37], first identified in vivo in sperm in the oviductal ampulla at the time of fertilization [24]. Hyperactivated movement is characterized as a series of higher-amplitude, asymmetrical flagellar bends [41,42,43,44], which are associated with an increment in the sperm’s curvilinear velocity and a decrease in linearity [40,41,45]. In murid rodents, hyperactivation is characterized by a “figure of eight” or whip-like movement [42,46]. Hyperactivated motility ensures that sperm can swim through the viscoelastic environment of the oviduct [43,47,48] and aids sperm penetration of oocyte vestments [42]. In vivo, hyperactivation takes place in response to specific signals that appear in the oviduct shortly before ovulation [49]. Several infertility phenotypes in mice and humans have been found to associate with defects in the development of hyperactivation [50,51,52,53], underscoring the relevance of this process during preparation for fertilization. Various studies have revealed that capacitation and hyperactivation could be dissociated [54], but it has not been conclusively demonstrated that they are completely independent processes [23,55].

In sperm cells, beating of the flagellum is produced through the hydrolysis of ATP catalysed by dynein ATPases in the axoneme [56,57]. Thus, sperm motility and velocity depend on the amount of ATP available [58,59]. In mammalian sperm, the synthesis of ATP takes place via two main metabolic pathways: oxidative phosphorylation (OXPHOS), which occurs in the midpiece mitochondria, and glycolysis, which takes place in the fibrous sheath associated with the principal piece [60,61,62]. Historically, OXPHOS has been regarded as the main source of ATP production for sperm motility with glycolysis, having a secondary role [63,64,65]. Nevertheless, the relative prevalence of each metabolic pathway in the generation of ATP for sperm motility is highly species-specific in mammals. Thus, there are species whose sperm have high respiration rates (boar and horse), species with high respiration and glycolytic rates (bull and guinea pig), and other species whose spermatozoa depend mainly on the glycolytic pathway (human) [66]. In the mouse, both metabolic pathways, glycolysis and OXPHOS, are essential to sustain vigorous sperm motility and fertility [30,33,67,68,69]. Mouse sperm exposed to respiratory inhibitors [70] or mitochondrial uncouplers [30,33] are unable to sustain ATP levels and progressive motility. In addition, studies in glyceraldehyde phosphate dehydrogenase (GAPDS) knockout mice [71], knockout mice for other glycolytic enzymes [72,73], or in sperm exposed to chemical inhibitors of glycolysis [33,74,75,76,77] showed that sperm motility and ATP production are negatively affected and that these sperm cells have decreased fertilizing capacity. This evidence thus indicates that OXPHOS and glycolysis are essential for mouse sperm when they swim towards the ovum and participate in fertilization.

The content of ATP in activated spermatozoa tends to decrease with time after ejaculation, producing a reduction in motility, in parallel to a decrease in flagellar beating frequency and velocity [60]. In addition, a series of processes that are essential to capacitation, such as hyperactivation [30,33], extensive protein phosphorylation [67], acrosome reaction [78], and fertilization [71,78,79], may impose energetic demands, in addition to those required for flagellar motility over relatively long periods of time. Studies in primates reported a decrease of intracellular ATP in sperm associated with hyperactivation, and this decrease was associated with increases in curvilinear velocity and flagellar beat frequency [80].

In the present study we examined variations in intracellular ATP levels when sperm cells from three mouse species (*Mus musculus*, *M. spretus*, and *M. spicilegus*) were incubated under capacitating conditions that are known to render sperm from these species capable of undergoing the acrosome reaction in response to a physiological stimulus [81]. In previous studies, we found that sperm from these species differ in sperm ATP content under basal conditions [73,82] and also in the pathways they use to generate ATP [59]. In addition, we assessed changes in motility, swimming patterns, and proportion of hyperactivated spermatozoa in the three species. We hypothesized that under capacitating conditions there would be a relationship between intracellular ATP levels and changes in motility and swimming patterns and that these would be associated with the development of hyperactivation.

## 2. Materials and Methods

### 2.1. Animals and Sperm Collection

Outbred adult males from three mouse species (*Mus musculus*, *M. spretus*, and *M. spicilegus*) were used in this study. The specimens were purchased from the Institut des Sciences de l`Evolution, CNRS-Université Montpellier 2, France, which maintained wild-derived colonies that have been kept in captivity for <30 generations. Sample size varied between 6 and 8 males for each species (*M. musculus* = 7 individuals, *M. spretus* = 8 individuals, and *M. spicilegus* = 6 individuals). Males were kept in our facilities in individual cages under controlled temperature (20–24 °C) on a 14 h light and 10 h darkness photoperiod. They were provided with water and food *ad libitum*. Animals 3–5 months old were sacrificed by cervical dislocation, which is regarded as a humane method by the Spanish and European regulations. No other procedures were involved.

Males were sacrificed, and both caudae epididymides were removed. Each cauda epididymis was placed in a 35 mm plastic culture dish containing a fixed volume of culture medium at 37 °C. The volume of the medium used was adjusted to obtain a concentration of 20 × 10^6^ cells/mL, according to total sperm numbers estimated for each species in previous studies [83]. One cauda epididymis was placed in non-capacitating medium (mT-H) under air and the other one was placed in capacitating medium (mT-BH) under 5% CO_2_/air. Composition of media was based on a modified Tyrode’s medium [34]. The non-capacitating medium mT-H had the following composition: 131.89 mN NaCl, 2.68 mM KCl, 0.49 mM MgCl_2_.2H_2_O, 0.36 mM NaH_2_PO_4_.2H_2_O, 20 mM Hepes, 5.56 mM glucose, 1.80 mM CaCl_2_, 5 µg phenol red/mL, 50 µg kanamycin/mL, and 4 mg bovine serum albumin/mL. The capacitating medium mT-BH differed from mT-H in that 15 mM NaHCO_3_ was added and NaCl was adjusted to 116.89 mM to maintain a similar osmolality [34]. The pH was adjusted to 7.4 in both media prior to incubation. The mT-H medium was maintained at 37 °C in air and mT-BH medium at 37 °C with 5% CO_2_/air. To collect spermatozoa, small incisions were performed in each cauda to allow cells to swim out. After 5 min, epididymal tissue was discarded and the sperm suspension was transferred to a plastic tube under a suitable atmosphere. This standard collection protocol, as used routinely in our laboratory [59,73,82], ensures the recovery of adequate sperm numbers and minimizes the time of exposure of sperm cells to incubation conditions before initial sperm assessments. Seminal parameters were evaluated immediately (0 min for the purpose of this study) and after 30, 60, and 90 min incubations. Sperm collection procedures and incubations were carried out at 37 °C.

### 2.2. Sperm Parameters

Sperm concentration in the different sperm suspensions was estimated using a modified Neubauer chamber and 100× magnification under phase contrast microscopy (Ci microscope, Nikon, Tokyo, Japan). The number of sperm per epididymis was calculated as sperm concentration × volume of the sperm suspension. Total sperm number for each male was the sum of the total number of sperm of the two epididymides. Sperm viability was assessed in sperm smears stained with eosin-nigrosin and Giemsa [84]. The sperm suspension (5 µL) was first mixed with 10 µL eosin-nigrosin solution on a glass slide, and the mix was smeared after 30 s at 37 °C. Subsequently, smears were stained with Giemsa solution and mounted with DePeX. The spermatozoa (100 per smear) were examined with a 100× objective under bright field microscopy (Ci microscope, Nikon, Madrid, Spain), and were considered viable if they excluded eosin.

Sperm motility was assessed placing 10 µL of the sperm suspension between a pre-warmed slide and coverslip. Percentage of motile sperm was assessed subjectively to the nearest 5%, at 100× magnification, under phase-contrast microscopy (Ci microscope, Nikon) by two independent observers, and the mean value of the two observations was used.

Sperm capacitation was assessed by staining cells with the vital stain Hoechst 33,258 bisbenzamide (B2883, Sigma, Madrid, Spain) to distinguish non-viable from viable cells and then with chlortetracycline (CTC) (C4881, Sigma) to identify several physiological patterns [74] (Figure S1). A volume of 100 µL of the sperm suspension was mixed with 50 µL of Hoechst 33,258 bisbenzamide (concentration 6 µg/mL) and incubated during 1 min in the dark. Subsequently, the samples were centrifuged for 2 min at 100× *g*, the supernatant discarded, the pellet resuspended in 100 µL of medium and fixed with a glutaraldehyde-sodium cacodylate solution (concentration 0.165 M). Immediately before evaluation, the cells were stained with CTC (concentration 250 µM) by adding 20 µL of CTC solution to 20 µL of sperm suspension and incubating in the dark for 3 min. For evaluation, spermatozoa in each sample were examined at 1000× magnification under fluorescence and phase contrast microscopy simultaneously (E-600 microscope, Nikon). Pre-fixation viability of the spermatozoa was assessed using a Nikon UV-2A 330-nm filter and fluorescence emission via a DM 400 dichroic mirror. Only the cells that excluded the Hoechst stain were considered viable. Capacitation status (CTC staining patterns) was evaluated in 100 viable spermatozoa (i.e., those excluding Hoechst 33258) per sample using a Nikon BV-2A 405-nm filter and fluorescence emission with a DM 455 dichroic mirror, distinguishing the following patterns: (a) F (non-capacitated sperm): the head of spermatozoa was uniformly stained with CTC; (b) B (capacitated sperm): the post-acrosomal region of the head was not stained with CTC; and (c) AR (without the sperm acrosome): the head of the sperm cell showed little or no CTC staining [85] (Figure S1).

For sperm swimming analysis, sperm suspensions were diluted with medium to obtain an approximate concentration of 4–6 × 10^6^ sperm/mL, and 6 µL of suspension was placed in a pre-warmed 20 µm chamber (Leja, Nieuw-Vennep, The Netherlands). Sperm parameters were evaluated using the program SCA v.5.0 (Microptic, Barcelona, Spain). A total of six random fields were recorded at 75 frames/s during 1 s using a video camera (Basler A312fc, Vision Tecnologies, Glen Burnie, MD, USA) at 40× magnification under negative phase contrast optics (Ci microscope, Nikon). Sperm swimming parameters were evaluated in ~200 spermatozoa per sample. The following kinetic parameters were measured: curvilinear velocity (µm/s) (VCL), straight-line velocity (µm/s) (VSL), linearity (%) (LIN = VSL/VCL), amplitude of lateral head displacement (µm) (ALH), and beat cross frequency (Hz) (BCF).

Sperm ATP content was determined using a luciferase-based ATP bioluminescent assay kit (ATP Bioluminiscence Assay Kit HS II, Roche Farma S.A., Madrid, Spain), as previously described [73,82,86]. Briefly, a diluted aliquot of sperm suspension was mixed in equal volumes with Cell Lysis Reagent, vortexed, and incubated at room temperature for 5 min. The resulting cell lysate was centrifuged at 12,000× *g* for 2 min, and 50 µL of supernatant was recovered and immediately frozen in liquid N_2_. Biolminescence was measured in triplicate in 96-well plates using a luminometer (BioTeK Sinergy HT, Winooski, VT, USA). A total of 50 µL of luciferase reagent was added to 50 µL of the sample (via auto-injection) and light emission was measured over a 5 s integration period. Standard bioluminescence curves were obtained for each plate by measuring solutions with known concentrations of ATP alongside the samples. The content of ATP per spermatozoa (amoles/sperm) was calculated, dividing the total ATP content per well by the number of sperm equivalent to the volume of sperm lysate in the well (mean ± SEM = 433 ± 41 cells well^−1^).

### 2.3. Percentage of Hyperactivation

**Figure 1:**
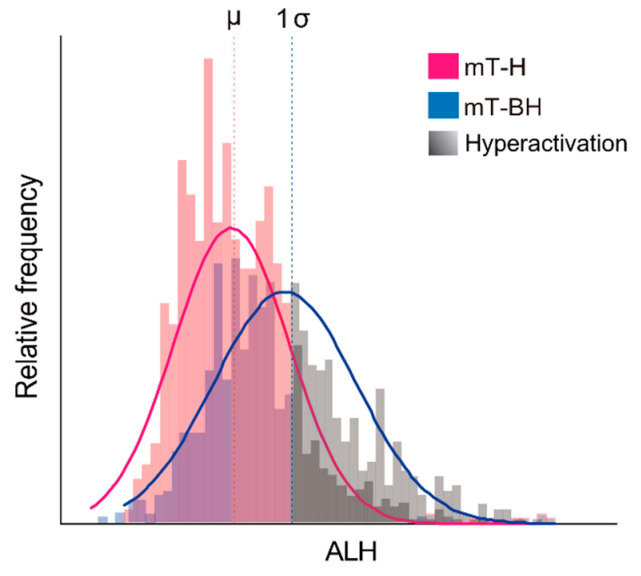
Example for the estimation of the percentage of hyperactivated spermatozoa in one individual of *M. musculus*. The relative frequency of values of amplitude of lateral head displacement (ALH) was plotted for spermatozoa, incubated under non-capacitating and capacitating conditions over time; see text for details. Non-capacitating conditions (mT-H medium): pink line and bars. Capacitating conditions (mT-BH medium): blue line and bars. Sperm in each condition are classified as hyperactivated when their ALH value surpasses the mean +1 standard deviation interval estimated from the control (non-capacitating) condition. Hyperactivated sperm are represented with different tones of grey. µ: ALH mean in non-capacitating conditions. 1σ: 1 standard deviation.

The percentage of hyperactivated sperm was estimated based on the measured swimming parameters. Frequency histograms were constructed for the kinematic parameters, and the swimming parameter that registered the greatest frequency change between treatments was selected as indicative of changes, occurring as a result of capacitation/hyperactivation. In our dataset, ALH was observed to increase over time under capacitating conditions (incubation in medium mT-BH), while remaining unchanged in the non-capacitating medium (mT-H). Other studies had previously observed the same behaviour, thus recommending the use of ALH as an indicator of sperm hyperactivation [42,87]. ALH distribution intervals were estimated for non-capacitating samples at 0, 30, 60, and 90 min of incubation using the mean ± 1 standard deviation as limits. Spermatozoa in the capacitation treatment were classified as hyperactivated when their ALH value exceeded the maximum limit (mean + 1 standard deviation) of ALH frequency distribution for the controls (i.e., noncapacitating medium) in their respective incubation time (see Figure 1 as a graphical example on one individual of *M. musculus*). Sperm with VAP < 20 µm s^−1^ were excluded from this analysis, both for the interval construction and classification. The rationale for this exclusion was that sperm with slow motility would fail to hyperactivate, thus resulting in an artificial underestimation of the percentages based on ALH variation.

### 2.4. Data Analysis

All variables were log_10_-transformed for statistical analyses, with the exception of percentages (viability, motility, linearity), which were arcsine-transformed. Repeated measures of analyses of variance (ANOVAs) were performed for each of the variables, using time (4 levels: 0, 30, 60, and 90 min) and treatment (2 levels: mT-H and mT-BH) as fixed factors and individual as a random factor. Additionally, a post-hoc Fisher LSD test was performed for each treatment at different times and for each time at different treatments. Pearson’s correlation was performed between the percentage of capacitated sperm and percentage hyperactivated sperm in capacitating conditions (mT-BH medium) All statistic tests were performed with IBM SPSS Statistics v20 at α = 0.05.

## 3. Results

### 3.1. Viability and Motility

**Figure 2:**
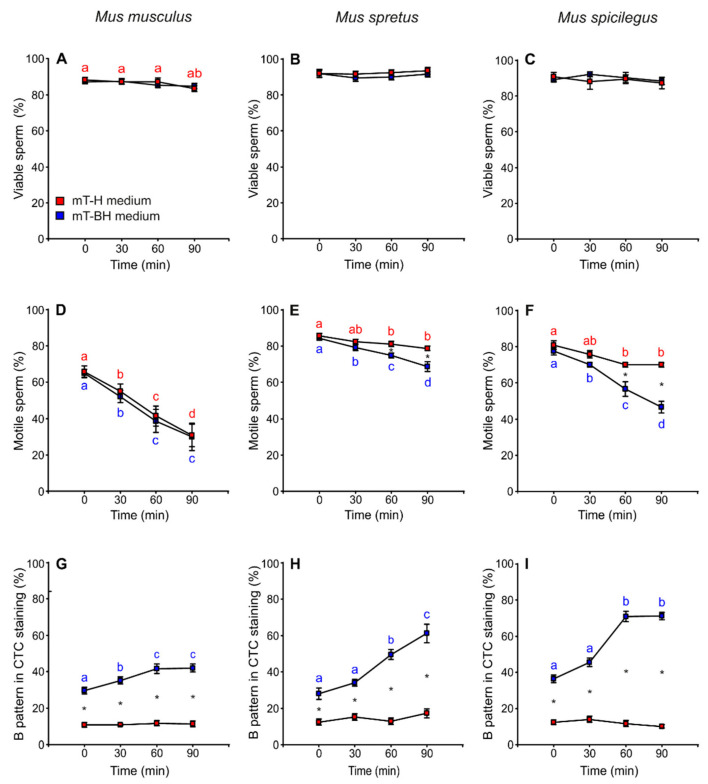
Variation in sperm viability, sperm motility, and capacitated sperm in *M. musculus*, *M. spretus* and *M. spicilegus* incubated under non-capacitating conditions (mT-H medium) and capacitating conditions (mT-BH medium). Data are means ± SE (*n* = 7 in *M. musculus*, *n* = 8 in *M. spretus* and *n* = 6 in *M. spicilegus*). Red squares: non-capacitating conditions, mT-H medium under air. Blue squares: capacitating conditions, mT-BH medium under 5% CO_2_/air. (**A**–**C**) Percentage of viable sperm. (**D**–**F**) Percentage of motile sperm (**G**–**I**) Percentage of B pattern in CTC staining (%). (**A**,**D**,**G**) *M. musculus*. (**B**,**E**,**H**) *M. spretus*. (**C**,**F**,**I**) *M. spicilegus*. Asterisks indicate significant differences (*p* < 0.05) between treatments for the same time in a Fisher post-hoc test. Different letters between times of incubation for the same treatment indicate statistically significant differences (*p* < 0.05) in a Fisher post-hoc test. If two time-points of an incubation share any letter, the differences between them are not significant. No letters were used when a time-point of an incubation did not show any significant effect in the dependent variable.

*Mus musculus* showed a slight decrease in percentage of live spermatozoa throughout time (*p* = 0.013), although there were no significant differences between spermatozoa incubated in mT-H or mT-BH (non-capacitating or capacitating conditions, respectively) (Figure 2A, Table 1). In *M. spretus*, viability was constant during incubation but showed slight and significant treatment-related differences (*p* = 0.037) (Figure 2B, Table 1). In *M. spicilegus*, the percentage of live sperm showed no significant differences throughout time or between media (Figure 2C, Table 1).

The percentage of motile spermatozoa showed a time-related decrease in both media in *M. musculus* (*p* < 0.001), and there were no significant differences between non-capacitating (mT-H) and capacitating (mT-BH) media (Figure 2D, Table 1). On the other hand, *M. spretus* and *M. spicilegus* sperm showed a decrease in the percentage of motility over time, which was more pronounced under capacitating conditions (*M. spretus*: treatment × time *p* = 0.004; *M. spicilegus*: treatment × time *p* < 0.001) (Figure 2E,F, Table 1).

### 3.2. Capacitation

The percentage of live spermatozoa exhibiting a B pattern when stained with chlortetracycline (indicative of capacitation; see Materials and Methods for details) was assessed over time in cells incubated in mT-H (non-capacitating conditions) and mT-BH (capacitating conditions). The percentage of cells with a B pattern remained roughly unchanged in spermatozoa incubated in mT-H (average values of 11% in *M. musculus*, 14% in *M. spretus*, and 12% in *M. spicilegus*) (Figure 2G–I, Table 1). On the other hand, the percentage of cells with a B pattern increased over time in spermatozoa from the three species incubated in mT-BH (*M. musculus:* treatment × time *p* = 0.032; *M. spretus*: treatment × time *p* < 0.001; *M. spicilegus*: treatment × time *p* = 0.022). There were species-specific differences with regards to the percentage of spermatozoa exhibiting the B pattern during incubation in mT-BH (Figure 2G–I, Table 1). *M. musculus* showed the lowest percentage of sperm with B pattern (41.6%), peaking at 60 min and remaining stable at 90 min (Figure 2G). *M. spretus* showed an intermediate percentage of B pattern (61.2%), reaching its maximum value at 90 min (Figure 2H). *M. spicilegus* samples had the highest percentage of B pattern (71%), which was observed after 60 min of incubation under capacitating conditions (Figure 2I).

The percentage of cells showing spontaneous acrosome reactions (as revealed by the AR pattern of staining with chlortetracycline; see Materials and Methods for details) was low (<20%) under both conditions in the three species. This measure tended to be rather constant throughout incubations, although it increased slightly at 60 min in sperm of *M. musculus* in both conditions (*p* < 0.001) and in sperm of *M. spicilegus* in non-capacitating conditions (*p* = 0.029) (Figure S2, Table S1).

### 3.3. Sperm Swimming Parameters

**Figure 3:**
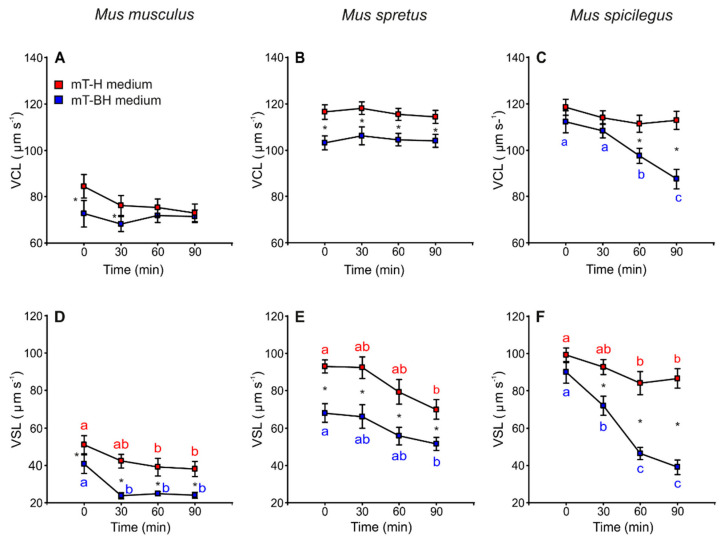
Variation in sperm swimming parameters (VCL and VSL) from three mouse species (*M. musculus*, *M. spretus*, *M. spicilegus*) incubated under non-capacitating conditions (mT-H medium) and capacitating conditions (mT-BH medium). Data are means ± SE (*n* = 7 in *M. musculus*, *n* = 8 in *M. spretus*, and *n* = 6 in *M. spicilegus*). Red squares: non-capacitating conditions, mT-H medium under air. Blue squares: capacitating conditions, mT-BH medium under 5% CO_2_/air. (**A**–**C**) Curvilinear velocity (VCL) (µm s^−1^). (**D**–**F**) Straight-line velocity (VSL) (µm s^−1^). (**A**,**C**) *M. musculus*. (**B**,**E**) *M. spretus*. (**C**,**F**) *M. spicilegus*. Asterisks indicate significant differences (*p* < 0.05) between treatments for the same time in a Fisher post-hoc test. Different letters between times of incubation for the same treatment indicate statistically significant differences (*p* < 0.05) in a Fisher post-hoc test. If two time-points of an incubation share any letter, the differences between them are not significant. No letters were used when a time-point of an incubation did not show any significant effect in the dependent variable.

The incubation in capacitating medium (mT-BH) showed sperm with slower velocity (VCL and VSL) in the three species. There were differences between species with regards to the decrease in sperm velocity during incubations. In *M. musculus*, VCL remained constant over time in mT-H (non-capacitating medium) and mT-BH (capacitating medium) (Figure 3A, Table 1). VSL exhibited a significant decrease (*p* < 0.001) at 30 min in mT-BH and a slight decrease at 60 min in mT-H medium (Figure 3D, Table 1). In the case of *M. spretus*, VCL remained stable over time in both media (Figure 3B, Table 1), while VSL values, in both media, showed the same pattern, decreasing steadily after 30 min (*p* < 0.001) (Figure 3E, Table 1). *M. spicilegus* sperm incubated in mT-H did not show significant differences in VCL over time but a slight decrease in VSL at 60 min (Figure 3C,F, Table 1). On the other hand, sperm incubated in capacitating conditions showed pronounced decreases in the two velocity descriptors over time (treatment × time *p* < 0.001) (Figure 3C,F, Table 1).

**Figure 4:**
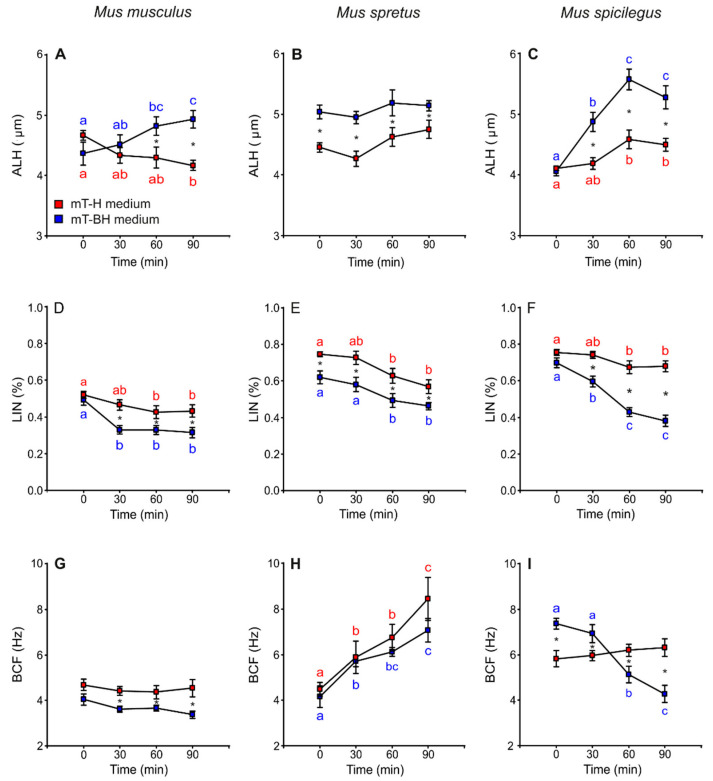
Variation in sperm swimming parameters (ALH, LIN and BCF) from three mouse species (*M. musculus*, *M. spretus*, *M. spicilegus*) incubated under non-capacitating conditions (mT-H medium) and capacitating conditions (mT-BH medium). Data are means ± SE (*n* = 7 in *M. musculus*, *n* = 8 in *M. spretus*, and *n* = 6 in *M. spicilegus*). Red squares: non-capacitating conditions, mT-H medium under air. Blue squares: capacitating conditions, mT-BH medium under 5% CO_2_/air. (**A**–**C**) Lateral head displacement (ALH) (µm). (**D**–**F**) Linearity (LIN) (VCL/VSL) (%). (**G**–**H**) Beat cross frequency (BCF) (Hz). (**A**,**D**,**G**) *M. musculus*. (**B**,**E**,**H**) *M. spretus.* (**C**,**F**,**I**) *M. spicilegus*. Asterisks indicate significant differences (*p* < 0.05) between treatments for the same time in a Fisher post-hoc test. Different letters between times of incubation for the same treatment indicate statistically significant differences (*p* < 0.05) in a Fisher post-hoc test. If two time-points of an incubation share any letter, the differences between them are not significant. No letters were used when a time-point of an incubation did not show any significant effect in the dependent variable.

Lateral head displacement (ALH) was the only variable that showed higher values in sperm incubated under capacitating conditions than in non-capacitating conditions. Spermatozoa from *M. musculus* and *M. spicilegus* incubated in mT-BH exhibited an increase in ALH values over time (*M. musculus:* treatment × time *p* = 0.001; *M. spicilegus*: treatment × time *p* = 0.003) (Figure 4A,C, Table 1). In *M. spretus*, ALH values were higher from the beginning of incubation in mT-BH than in mT-H (*p* < 0.001) and remained so throughout incubation in this medium (Figure 4B, Table 1).

In *M. musculus*, linearity (LIN) remained constant over time, with the exception of a sharp decrease at 30 min with regards to time 0 in samples incubated in capacitating medium (*p* < 0.001) (Figure 4D, Table 1). In *M. spretus,* the values for samples under capacitating conditions were significantly lower than those of the non-capacitating samples at all incubation times (*p* < 0.001). Both treatments showed a significant decrease after 60 min of incubation (*p* < 0.001) (Figure 4E, Table 1). In *M. spicilegus*, samples incubated under non-capacitating conditions exhibited fairly constant LIN values over time, with a slight decline at 60 min. Sperm incubated in capacitating medium showed decreasing values during incubation (treatment × time *p* < 0.001) (Figure 4F, Table 1).

*M. musculus* sperm showed stable values of beat cross frequency (BCF) over time in both media, although the values in mT-BH were lower than those in mT-H (*p* < 0.001) (Figure 4G, Table 1). In *M. spretus,* BCF increased over time with no significant differences between mT-H and mT-BH (*p* < 0.001) (Figure 4H, Table 1). In *M. spicilegus*, BCF showed stable values over time in mT-H and a time-related decrease in spermatozoa incubated in mT-BH (treatment × time *p* < 0.001) (Figure 4I; Table 1).

### 3.4. ATP Content

**Figure 5:**
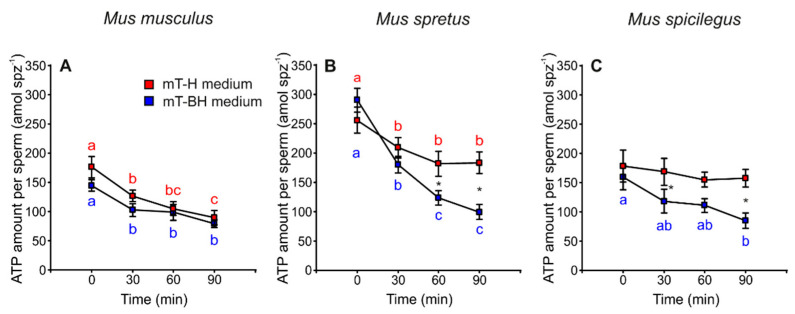
Variation in ATP amount per sperm in three mouse species (*M. musculus*, *M. spretus*, *M. spicilegus*) incubated under non-capacitating conditions (mT-H medium) and capacitating conditions (mT-BH medium). Data are means ± SE (*n* = 7 in *M. musculus*, *n* = 8 in *M. spretus*, and *n* = 6 in *M. spicilegus*). Red squares: non-capacitating conditions, mT-H medium under air. Blue squares: capacitating conditions mT-BH medium under 5% CO_2_/air. (**A**–**C**) ATP amount per sperm (amol spz^−1^). (**A**) *M. musculus*. (**B**) *M. spretus*. (**C**) *M. spicilegus*. Asterisks indicate significant differences (*p* < 0.05) between treatments for the same time in a Fisher post-hoc test. Different letters between times of incubation for the same treatment indicate statistically significant differences (*p* < 0.05) in a Fisher post-hoc test. If two time-points of an incubation share any letter, the differences between them are not significant. No letters were used when a time-point of an incubation did not show any significant effect in the dependent variable.

The results revealed a decrease in ATP content over time in spermatozoa incubated in mT-H and mT-BH in the three species (Figure 5A–C, Table 1). In *M. musculus*, ATP content decreased significantly and with similar intensity under both conditions (*p* < 0.001) (mT-H and mT-BH media). On the other hand, *M. spretus* and *M. spicilegus* showed different trends when incubated under different conditions (mT-H or mT-BH media). In *M. spretus*, ATP content decreased in both treatments over time, but it decreased more quickly under capacitating conditions (treatment × time *p* = 0.001). In *M. spicilegus*, ATP content only decreased over time in the capacitating medium, while remaining stable in the non-capacitating medium (treatment × time *p* = 0.016).

**Figure 6:**
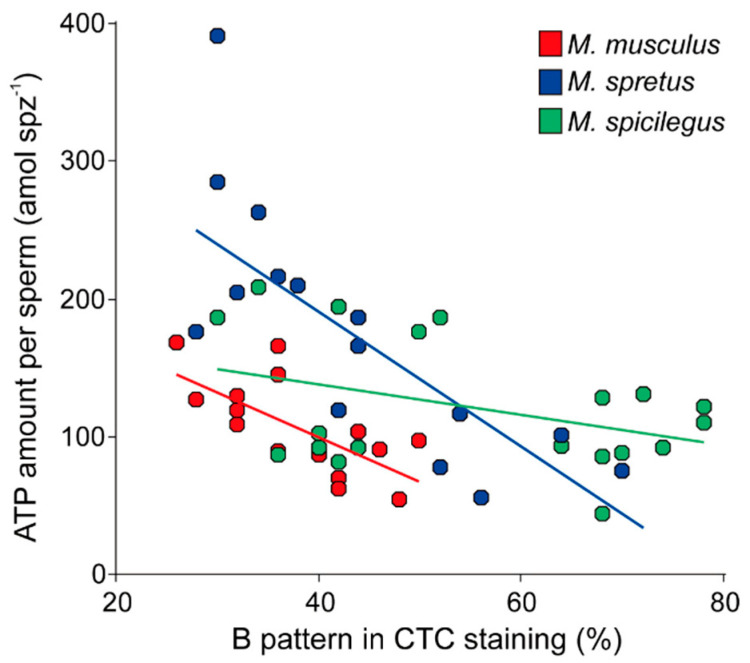
Association between percentage of cells with B pattern after chlortetracycline (CTC) staining, indicative of sperm capacitation, and sperm ATP content in three mouse species (*M. musculus*, *M. spretus*, *M. spicilegus*) incubated in capacitating conditions (mT-BH medium). Each datapoint corresponds to a single incubation time/experiment combination.

In order to further explore the relationships between capacitation and ATP content in these species, we performed regression analyses for each species using a mixed linear model with ATP content under capacitating conditions (log-transformed) as the dependent variable, a proportion of capacitated sperm (arcsine-transformed) as the fixed predictor, and individual as a random factor. These tests resulted in negative and significant associations between the percentage of capacitated sperm and samples ATP content in the three species (*M. musculus*: intercept = 2.85, slope = −1.27, *p* = 0.008; *M. spretus*: intercept = 3.10, slope = −1.27, *p* < 0.001; *M. spicilegus*: intercept = 2.40, slope = −0.40, *p* = 0.039) (Figure 6). We then compared the regression slopes via a fourth model that added the species and its interaction with the proportion of capacitated sperm, as second and third predictors respectively. The three predictors were significantly associated with the amount of ATP per sperm in the samples (proportion of capacitated cells: *F* = 29.25, *p* < 0.0001; species: *F* = 5.47, *p* = 0.0078; interaction: *F* = 5.91, *p* = 0.0058). The significant interaction between the two independent variables meant that the slopes of the regressions differed between species, which in turn indicated that the decrease in ATP content in sperm, associated with the incubation in capacitating conditions, occurs in a species-specific manner.

### 3.5. Hyperactivation

**Figure 7:**
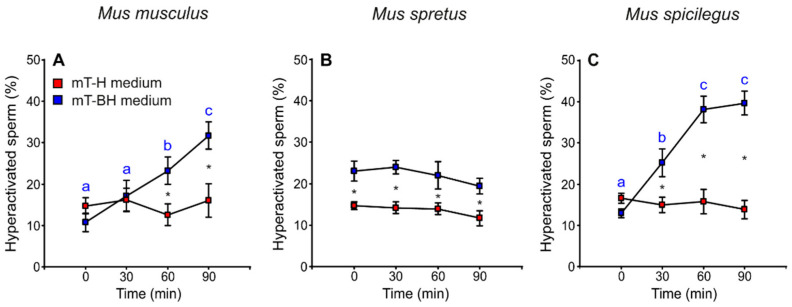
Variation in the proportion of hyperactivated sperm during incubation of sperm cells from three mouse species (*M. musculus*, *M. spretus*, *M. spicilegus*) under non-capacitating conditions (mT-H medium) and capacitating conditions (mT-BH medium). Data are means ± SE (*n* = 7 in *M. musculus*, *n* = 8 in *M. spretus*, and *n* = 6 in *M. spicilegus*). Red squares: non-capacitating conditions, mT-H medium under air. Blue squares: capacitating conditions, mT-BH medium under 5% CO_2_/air. (**A**–**C**) Percentage of hyperactivated sperm (%). (**A**) *M. musculus*. (**B**) *M. spretus*. (**C**) *M. spicilegus*. Asterisks indicate significant differences (*p* < 0.05) between treatments for the same time in a Fisher post-hoc test. Different letters between times of incubation for the same treatment indicate statistically significant differences (*p* < 0.05) in a Fisher post-hoc test. If two time-points of an incubation share any letter, the differences between them are not significant. No letters were used when a time-point of an incubation did not show any significant effect in the dependent variable.

The proportion of sperm cells with hyperactivated motility was calculated, taking into account sperm swimming patterns (see Materials and Methods). The amplitude of lateral head displacement (ALH) exhibited the greatest frequency change between sperm incubated under non-capacitating and capacitating conditions and thus served as a marker for hyperactivation. The percentage of hyperactivated cells increased over time in *M. musculus* and *M. spicilegus* sperm incubated in mT-BH (capacitating medium) (*M. musculus*: treatment × time *p* < 0.001; *M. spicilegus*: treatment × time *p* < 0.001), with values reaching a maximum at 90 min (32% hyperactivated sperm in *M. musculus* and 40% in *M. spicilegus*). Spermatozoa incubated in mT-H (non-capacitating medium) exhibited low and stable values of hyperactivated sperm over time in these two species (16% in *M. musculus* and 15% in *M. spicilegus*) (Figure 7A,C, Table 1). In *M. spretus*, the percentage of hyperactivation under non-capacitating conditions (mT-H) was also low (16%) and not different from that seen in the other two species (Figure 7B, Table 1). On the other hand, when *M. spretus* sperm were placed in capacitating conditions (medium mT-BH), the percentage of sperm classified as hyperactivated (22%) was significantly higher than that seen in non-capacitating controls at the beginning of incubation (*p* < 0.001) and remained so, without increasing further, during incubation (Figure 7B, Table 1).

Significant positive correlations were observed between the percentages of sperm cells exhibiting a capacitation pattern, estimated by staining with chlortetracycline, and those exhibiting a pattern of hyperactivation in *M. musculus* (*p* = 0.008) and *M. spicilegus* (*p* = 0.043) (Table 2). These results suggest that the processes of capacitation and hyperactivation are highly correlated and temporally associated in these two species.

### 3.6. Comparisons of Non-hyperactivated and Hyperactivated Subpopulations

**Figure 8:**
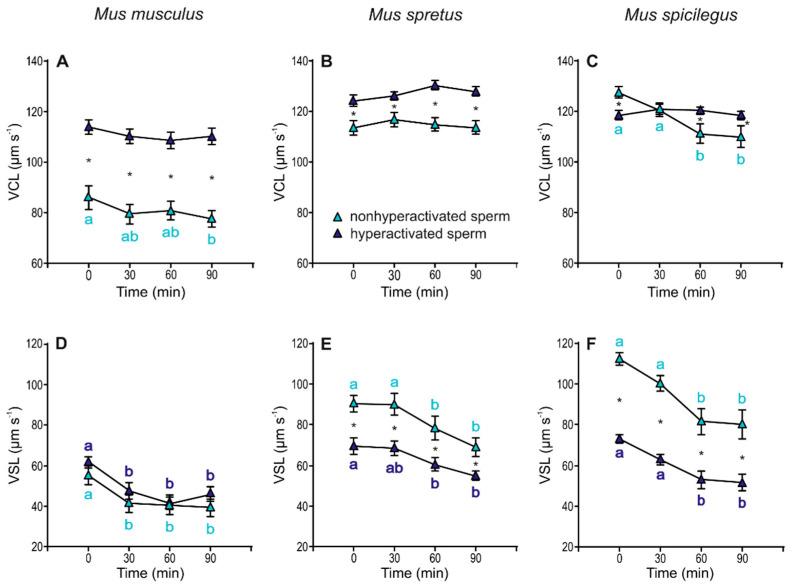
Variation in velocity descriptors (VCL and VSL) in hyperactivated and non-hyperactivated spermatozoa from three mouse species (*M. musculus*, *M. spretus*, *M. spicilegus*) under capacitating conditions (mT-BH medium under 5% CO_2_/air). Data are means ± SE (*n* = 7 in *M. musculus*, *n* = 8 in *M. spretus*, and *n* = 6 in *M. spicilegus*). Light blue triangles: non-hyperactivated sperm in capacitating conditions. Dark blue triangles: hyperactivated sperm in capacitating conditions. (**A**–**C**) Curvilinear velocity (VCL) (µm s^−1^), (**D**–**F**) Straight-line velocity (VSL) (µm s^−1^). (**A**,**D**) *M. musculus*. (**B**,**E**) *M. spretus*. (**C**,**F**) *M. spicilegus*. Asterisks indicate significant differences (*p* < 0.05) between non-hyperactivated and hyperactivated sperm for the same time in a Fisher post-hoc test. Different letters between times of incubation for the same status (hyperactivated or non-hyperactivated) indicate statistically significant differences (*p* < 0.05) in a Fisher post-hoc test. If two time-points of an incubation share any letter, the differences between them are not significant. No letters were used when a time-point of an incubation did not show any significant effect in the dependent variable.

Sperm incubated under capacitating conditions were analysed based on their ALH and classified into two subpopulations, non-hyperactivated and hyperactivated, and then sperm swimming descriptors were assessed for each subpopulation. Sperm in the hyperactivated subpopulation showed significantly higher VCL values than non-hyperactivated cells in the three species (Figure 8A–C, Table 3). These differences were evident at the beginning of incubation (0 min) in *M. musculus* (*p* < 0.001) and *M. spretus* (*p* < 0.001) and after 60 min of incubation in *M. spicilegus* (status × time *p* = 0.002). The values of VSL in hyperactivated and non-hyperactivated sperm decreased over time in the three species (*M. musculus*: *p* < 0.001; *M. spretus*: *p* < 0.001; *M. spicilegus*: *p* < 0.001) (Figure 8D–F), with significantly lower VSL values in hyperactivated sperm from *M. spretus* (*p* < 0.001) and *M. spicilegus* (*p* < 0.001) throughout the incubation time (Figure 8E,F, Table 3).

**Figure 9:**
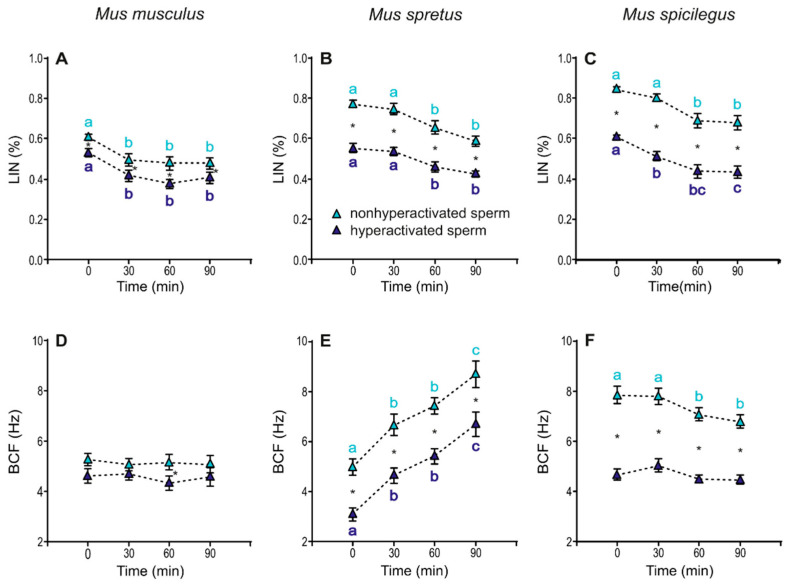
Variation in swimming parameters (LIN and BCF) of spermatozoa from three mouse species (*M. musculus*, *M. spretus*, *M. spicilegus*) under capacitating conditions (mT-BH medium under 5% CO_2_/air). Data are means ± SE (*n* = 7 in *M. musculus*, *n* = 8 in *M. spretus*, and *n* = 6 in *M. spicilegus*). Light blue triangles: non-hyperactivated sperm in capacitating conditions. Dark blue triangles: hyperactivated sperm in capacitating conditions. (**A**–**C**) Linearity (LIN) (VCL/VSL) (%). (**D**–**F**) Beat cross frequency (BCF) (Hz). (**A**,**D**) *M. musculus*. (**B**,**E**) *M. spretus*. (**C**,**F**) *M. spicilegus*. Asterisks indicate significant differences (*p* < 0.05) between non-hyperactivated and hyperactivated sperm for the same time in a Fisher post-hoc test. Different letters between times of incubation for the same status (hyperactivated or non-hyperactivated) indicate statistically significant differences (*p* < 0.05) in a Fisher post-hoc test. If two time-points of an incubation share any letter, the differences between them are not significant. No letters were used when a time-point of an incubation did not show any significant effect in the dependent variable.

Linearity (LIN) exhibited a slight (~10%) but significant decrease over time in both non-hyperactivated and hyperactivated sperm in the three species (*M. musculus*: *p* < 0.001; *M. spicilegus*: *p* < 0.001; *M. spicilegus*: *p* < 0.001) and was always higher in non-hyperactivated sperm (*M. musculus*: *p* < 0.001; *M. spretus*: *p* < 0.001; *M. spicilegus*: *p*< 0.001) (Figure 9A–C, Table 3). Beat cross frequency (BCF) values in *M. musculus* were stable over time and slightly but significantly higher between hyperactivated and non-hyperactivated sperm (*p* = 0.005) (Figure 9D, Table 3). In *M. spretus*, BCF increased over time in both sperm populations (*p* < 0.001), but non-hyperactivated sperm showed significantly higher values that hyperactivated sperm throughout time (*p* < 0.001) (Figure 9E, Table 3). In *M. spicilegus*, BFC had a slight decrease over time in non-hyperactivated sperm (*p* = 0.008), with hyperactivated sperm exhibiting stable and significantly lower values than non-hyperactivated sperm (*p* < 0.001) (Figure 9F, Table 3).

**Table 3:** Effect of time of incubation (“Time”), sperm status (“Status”: hyperactivated vs. non-hyperactivated), and interactions between time and status (“Interaction”) on kinetic parameters in three mouse species (*M. musculus*, *M., spretus*, and *M. spicilegus*) incubated under capacitating conditions (mT-BH medium). Values *F* and *p* correspond to statistical analyses of repeated measure ANOVAs. Individual was used as a random factor, and incubation conditions and time were used as fixed factors. Results in bold indicate significant differences (*p* < 0.05) in statistical analyses. VCL: curvilinear velocity; VSL: straight-line velocity; LIN: linearity; ALH: amplitude of lateral head displacement; BCF: beat-cross frequency.

Dependent Variable	Independent Variable	*Mus musculus*		*M. spretus*		*M. spicilegus*	
		*F*	*p*	*F*	*p*	*F*	*p*
VCL	Status	**215.217**	**<0.001**	**59.995**	**<0.001**	1.507	0.223
	Time	1.856	0.142	0.921	0.433	**5.03**	**0.003**
	Interaction	0.376	0.771	0.773	0.511	**5.361**	**0.002**
VSL	Status	3.589	0.061	**37.789**	**<0.001**	**111.676**	**<0.001**
	Time	**9.063**	**<0.001**	**8.13**	**<0.001**	**16.04**	**<0.001**
	Interaction	0.279	0.841	0.303	0.823	0.771	0.513
LIN	Status	**20.994**	**<0.001**	**125.778**	**<0.001**	**179.422**	**<0.001**
	Time	**13.285**	**<0.001**	**17.541**	**<0.001**	**17.741**	**<0.001**
	Interaction	0.15	0.929	0.519	0.670	0.603	0.698
ALH	Status	**2409.089**	**<0.001**	**2132.668**	**<0.001**	**2254.932**	**<0.001**
	Time	1.792	0.154	**5.758**	**0.001**	**30.844**	**<0.001**
	Interaction	0.726	0.539	**6.662**	**<0.001**	0.129	0.943
BCF	Status	**8.341**	**0.005**	**63.394**	**<0.001**	**215.774**	**<0.001**
	Time	0.168	0.917	**37.874**	**<0.001**	**4.18**	**0.008**
	Interaction	0.268	0.849	0.018	0.997	1.772	0.128

## 4. Discussion

The results of our study show that capacitation promotes changes in sperm swimming patterns and bioenergetics in three mouse species of the genus *Mus* (*M. musculus*, *M. spretus*, and *M. spicilegus*), and that these changes vary in extent and time of occurrence in a species-specific manner. In addition, results show differences between species with regards to sperm motility and ATP levels under non-capacitating conditions, but sperm parameters remained fairly unchanged over time in the three species under these conditions.

*M. musculus*, *M. spretus*, and *M. spicilegus* constitute a closely related monophyletic group of species with an estimated divergence time of approximately 1.7 million years [88]. Our previous comparative studies have revealed major differences between rodent species, including these mouse species, regarding relative testes mass, sperm numbers, percentage of motile sperm, swimming velocity, ATP content, and pathways for ATP generation, which have been associated with interspecific differences in sperm competition levels [59,81,82,83,84]. The differences in sperm parameters were observed among species in cells recently collected from the epididymis [83] and after incubation in conditions that would sustain sperm survival but not capacitation. These incubations revealed differences between species in the proportion of motile spermatozoa over time [59], a phenomenon that was also observed in this study. A relationship between the proportion of motile sperm and the amount of ATP among species was also evident [59,82]. Because spermatozoa from the three mouse species studied here maintained their viability throughout incubation time, it is most likely that the decrease in proportion of motile sperm is due, to a great extent, to differences in ATP availability [47] and also to the efficiency of ATP usage [89]. Among the mouse species examined here, *M. musculus* exhibited the greatest decrease in proportion of motile sperm, which agrees with the lowest amount of ATP in sperm cells throughout incubation.

The present results show that both the maximum percentage of capacitated cells and the time at which this maximum is reached also differ among these species, in agreement with what theory would predict from their levels of sperm competition [81]. Thus, the species with the highest sperm competition level (*M. spicilegus*) exhibited the highest rates and the earliest timing of capacitation and hyperactivation. On the other hand, the species with the lowest sperm competition level (*M. musculus*) was the species with the lowest rates and peaking later in performance. Observations in a different mouse genus (*Apodemus*) revealed similar trends in processes associated with capacitation when a species with high sperm competition levels (*A. sylvaticus*) was compared to another species with low sperm competition levels (*A. microps*). In this case, *A. sylvaticus* showed higher percentages of sperm with high flagellar tyrosine phosphorylation and spontaneous acrosome reaction, and a faster progression of these events through the sperm population than those seen in *A. microps* [90,91]. Altogether, these results suggest variation in the strategies evolved by species with regards to timing and efficacy of fertilization.

Not all capacitated sperm are able to fertilize since sperm must also maintain motility to reach the site of fertilization and to traverse the ovum vestments. Previous studies in laboratory mice have reported a detrimental effect of the process of capacitation on sperm motility [23,24,37,38,42,55]. Two of the three species analysed in our study (*M. spretus* and *M. spicilegus*) showed a decrease in motility when sperm were incubated under capacitating conditions. On the other hand, *M. musculus* did not show differences in the proportion of motile cells between the two incubation conditions, probably because over time there was also a decrease in motility under non-capacitation conditions.

The process of capacitation is also associated with changes in sperm swimming patterns (velocity and shape of sperm trajectories) [24], particularly an increase in the amplitude of the flagellar bend [24,41,43,44]. Since the head of the sperm moves from side to side, as a consequence of the principal and reverse bends, the amplitude of lateral head displacement (ALH) is an indirect measure of flagellar bend amplitude. In addition, previous studies [40,41,45] have shown that the convoluted trajectory shapes produced after incubation in capacitating medium are associated with an increase in VCL, which in time produces lower LIN scores. In our study, the sperm incubated under capacitating conditions showed higher ALH and lower LIN values than sperm incubated under non-capacitating conditions for the three species analysed. Nonetheless, the average VCL values of the sperm population in the capacitation treatment were lower than those in the non-capacitation treatment. This apparent incongruency was clarified when we separately analysed the subpopulations of hyperactivated and non-hyperactivated sperm among those incubated under capacitating conditions. Thus, hyperactivated sperm showed an increased preponderance of the lateral components of movement (increased ALH, VCL) over progressive components (decreased VSL, LIN) in the three species. The differences observed between both analyses suggest that the identification of the subpopulation that completed the hyperactivation process is crucial to provide accurate assessments of variations in motility patterns over time.

The three species analysed showed an increase in the percentage of hyperactivated sperm under capacitating conditions, and their pattern of hyperactivated movement showed an increased amplitude and similar/decreased velocity of lateral head movement (as evidenced by their lower BCF). However, the reduction in linearity of trajectory, a trait that was apparently common among the three species, resulted from different alterations of the movement pattern in each case. The hyperactivated sperm of *M. musculus* decreased their LIN by greatly increasing their VCL while maintaining VSL values similar to those of non-hyperactivated cells. In the case of *M. spretus*, the decrease of LIN associated with hyperactivation resulted from moderate changes in both parameters (increased VCL and decreased VSL). Lastly, *M. spicilegus* hyperactivated sperm showed lower LIN scores due to a slight increase in VCL accompanied by a strong decrease in VSL.

Previous studies have established sperm hyperactivation as a useful capacitation marker that can be identified prior to the acrosome reaction [92]. In coincidence with previous studies in laboratory mice [23,55], our results showed significant associations between increases in percentages of capacitated and hyperactivated sperm in *M. musculus* and *M. spicileugus*. However, *M. spretus* presented a rather different behaviour: while the percentage of capacitated cells increased over time, the percentage of hyperactivation remained stable. These results would suggest that in *M. spretus*, the phenomena of capacitation and hyperactivation may be dissociated over time. Alternatively, this discrepancy could indicate that the variation in ALH may not be a good parameter to define hyperactivation in *M. spretus*. The latter possibility seems improbable because ALH values differed between incubation conditions and the general swimming pattern of sperm incubated under capacitating conditions is coherent with previous definitions of hyperactivated movement. Thus, further studies are required to characterize the pattern of hyperactivation in this species.

Sperm motility is completely dependent on the availability of energy obtained from the enzymatic decomposition of ATP [58,73]. It seems reasonable to hypothesize that capacitation would impose an increase in energetic demands on the sperm cell as a consequence of changes in ion regulation, activation of intracellular signalling pathways (e.g., cAMP-PKA), or protein phosphorylation [27,30,33,35,36,79,93,94,95], which appears to be met by an increase in glucose uptake and consumption [96,97]. Moreover, changes in sperm propulsive versus transversal force ratio promoted by hyperactivation [98] could imply fundamental modifications of sperm ATP generation and usage. This appears to be at least partially supported by our results, since there was an increase in the proportion of capacitated cells together with a significantly faster decline of ATP levels in sperm of *M. spretus* and *M. spicilegus*. These species have the highest differences in ATP content between non-capacitated and capacitated sperm, as well as the highest differences in kinetic parameters associated with hyperactivation (VCL, VSL, LIN, ALH, and BCF). In addition, the species with the highest sperm competition level also has the highest ATP content, but the amount of ATP decreases more rapidly. These findings could thus be indicative of the deviation in the balance between sperm ATP consumption and production [73,89]. This imbalance could occur in three ways: (a) ATP production could remain stable while consumption increases, (b) ATP production could decrease while consumption remains stable or increases, and (c) ATP production and consumption could increase in different proportions, the consumption increasing more quickly than production. Further studies are required to discriminate between these options.

## 5. Conclusions

This study has revealed clear differences in the timing of capacitation and hyperactivation between species together with variation in the levels of ATP. Differences in the kinetics of capacitation or hyperactivation and in the proportion of cells that complete these processes could relate to diversity in how these species undergo such processes and their underlying regulatory mechanisms. Selection by forces such as sperm competition may have resulted in sperm from different species undergoing one or more of the processes required to participate in fertilization at different rates or paces. Therefore, this represents a relevant model to further understand subtleties in these processes and the mechanisms underlying them.

## Figures and Tables

**Table 1:** Effect of time of incubation (“Time”), incubation conditions (“Treatment”: non-capacitating vs. capacitating), and interactions between time and treatment (“Interaction”) on viability, motility, percentage of capacitated cells, velocity parameters, ATP content, and hyperactivation in spermatozoa from *M. musculus*, *M. spretus*, and *M. spicilegus*. Values *F* and *p* correspond to repeated measure ANOVAs. Individual was used as a random factor, and incubation conditions and time were used as fixed factors. Results in bold show significant differences (*p* < 0.05) in statistical analyses. VCL: curvilinear velocity; VSL: straight-line velocity; LIN: linearity; ALH: amplitude of lateral head displacement; BCF: beat-cross frequency.

Dependent Variable	Independent Variable	*Mus musculus*		*M. spretus*		*M. spicilegus*	
		*F*	*p*	*F*	*p*	*F*	*p*
Viability	Treatment	0.083	0.775	**4.609**	**0.037**	0.342	0.563
	Time	**4.059**	**0.013**	0.887	0.455	0.371	0.774
	Interaction	0.722	0.545	0.616	0.608	0.357	0.784
Motility	Treatment	0.430	0.515	**42.760**	**<0.001**	**50.392**	**<0.001**
	Time	**46.125**	**<0.001**	**32.808**	**<0.001**	**32.820**	**<0.001**
	Interaction	0.091	0.965	**5.113**	**0.004**	**7.858**	**<0.001**
VCL	Treatment	**9.589**	**0.003**	**44.188**	**<0.001**	**43.855**	**<0.001**
	Time	1.565	0.212	0.616	0.608	**12.069**	**<0.001**
	Interaction	1.326	0.279	0.204	0.893	**5.682**	**0.003**
VSL	Treatment	**46.589**	**<0.001**	**38.894**	**<0.001**	**102.109**	**<0.001**
	Time	**10.256**	**<0.001**	**7.257**	**<0.001**	**26.792**	**<0.001**
	Interaction	0.790	0.506	0.251	0.860	**9.035**	**<0.001**
LIN	Treatment	**29.628**	**<0.001**	**27.362**	**<0.001**	**115.201**	**<0.001**
	Time	**13.164**	**<0.001**	**10.630**	**<0.001**	**28.892**	**<0.001**
	Interaction	1.959	0.135	0.140	0.935	**9.492**	**<0.001**
ALH	Treatment	**10.003**	**0.003**	**33.020**	**<0.001**	**39.547**	**<0.001**
	Time	0.492	0.690	2.510	0.070	**21.088**	**<0.001**
	Interaction	**6.660**	**0.001**	0.341	0.796	**5.557**	**0.003**
BCF	Treatment	**26.949**	**<0.001**	1.918	0.172	0.225	0.638
	Time	0.831	0.484	**17.848**	**<0.001**	**9.697**	**<0.001**
	Interaction	0.665	0.578	0.596	0.620	**16.935**	**<0.001**
ATP content	Treatment	2.630	0.116	**11.309**	**0.002**	**10.243**	**0.003**
	Time	**13.823**	**<0.001**	**36.281**	**<0.001**	1.252	0.306
	Interaction	0.456	0.715	**6.299**	**0.001**	**1.802**	**0.016**
Capacitation (%)	Treatment	**94.367**	**<0.001**	**112.084**	**<0.001**	**88.798**	**<0.001**
	Time	1.713	0.187	**13.238**	**<0.001**	2.769	0.060
	Interaction	**1.223**	**0.032**	**9.270**	**<0.001**	**3.772**	**0.022**
Hyperactivation (%)	Treatment	**12.580**	**0.001**	**36.478**	**<0.001**	**56.084**	**<0.001**
	Time	**7.864**	**<0.001**	1.304	0.272	**10.314**	**<0.001**
	Interaction	**7.462**	**<0.001**	0.103	0.958	**13.293**	**<0.001**

**Table 2:** Pearson correlations between percentages of capacitated sperm and of hyperactivated sperm under capacitating conditions (mT-BH medium) in three mouse species (*M. musculus*, *M. spretus*, and *M. spicilegus*). Results in bold indicate significant differences (*p* < 0.05) in statistical analyses.

Species	*r_xy_*	*p*
*Mus musculus*	**0.99**	**0.0084**
*Mus spretus*	−0.04	0.8399
*Mus spicilegus*	**0.42**	**0.0426**

## Data Availability

Data are included in the article.

## Supplementary Materials

The following are available online at https://www.mdpi.com/article/10.3390/cells11020220/s1, Figure S1: Hoechst 33258 and chlortetracycline (CTC) staining patterns of mouse spermatozoa incubated under non-capacitating or capacitating conditions. Figure S2: Changes in chlortetracycline staining patterns in *M. musculus*, *M. spretus*, and *M. spicilegus* incubated under non-capacitating conditions or capacitating conditions. Table S1: Effect of time of incubation and incubation under non-capacitating or capacitating conditions on the percentage of spermatozoa, showing AR or F patterns after chlortetracycline staining in *M. musculus*, *M. spretus*, and *M. spicilegus*.
