# Supplementary material for: Energy Metabolism and Hyperactivation of Spermatozoa from Three Mouse Species under Capacitating Conditions
Source: Cells. 2022 Jan 10;11(2):220. doi: 10.3390/cells11020220 (PMC8773617; doi:10.3390/cells11020220)
Supplement: Supplementary file 1 [file cells-11-00220-s001.zip › cells-1498277-supplementary.pdf]

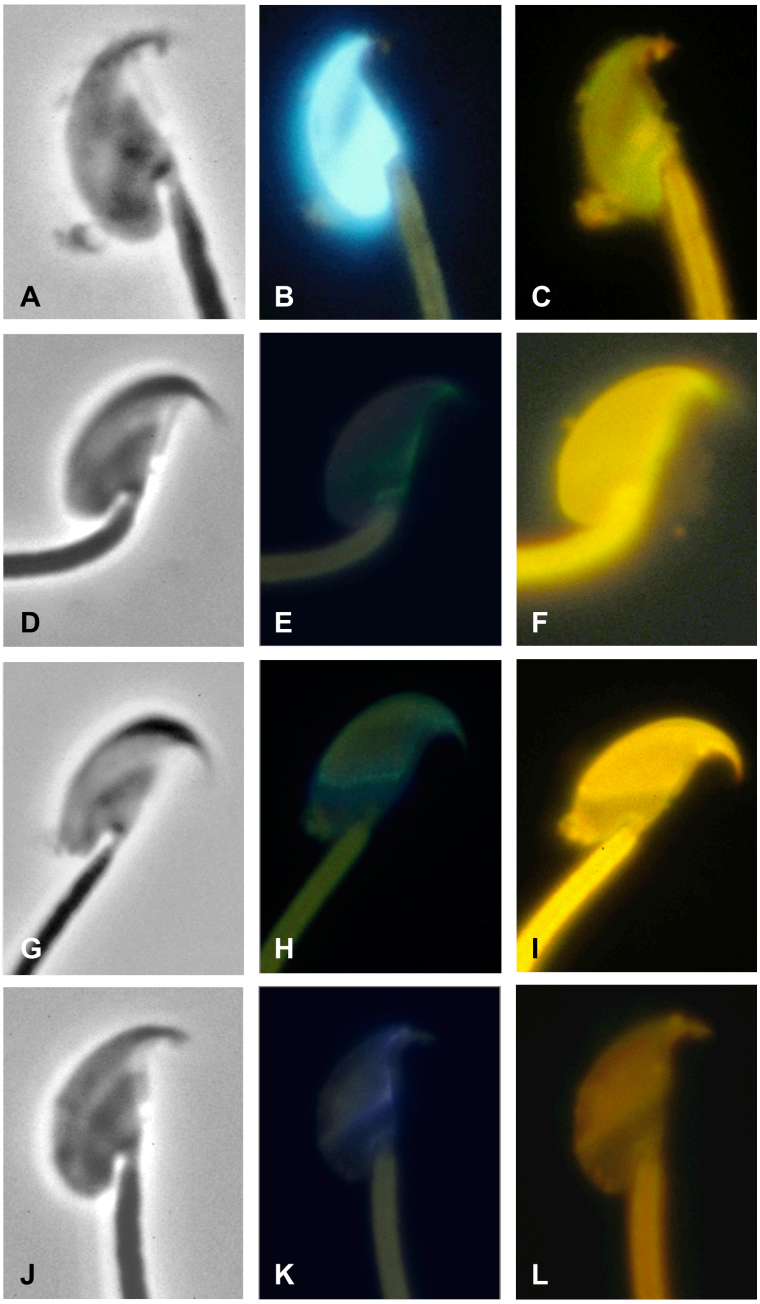

Figure S1. Hoechst 33258 and chlortetracycline (CTC) staining patterns of mouse spermatozoa incubated under noncapacitating or capacitating conditions. The figure depicts *Mus spretus* sperm cells; similar patterns are observed in the three species examined in this study. Spermatozoa were stained with Hoechst 33258 and with CTC at different times during incubation and examined under phase contrast and fluorescence microscopy. For details see Materials and Methods. (A-C) Nonviable (dead) spermatozoa. (D-F) Viable (live) noncapacitated spermatozoa. (G-I) Viable (live) capacitated spermatozoa. (J-L) Viable (live) sperm cells that have lost the acrosome spontaneouly during incubation. (A,D,G,J) Spermatozoa examined using phase contrast. (B,E,H,K) Spermatozoa examined under fluorescence employing a UV-2A filter (Nikon). (B) Sperm exhibiting staining with Hoechst 33258 and, hence, is not viable. (E,H,K) Sperm are not stained with Hoechst 33258 and, hence, are viable. (C,F,I,L) Spermatozoa examined under fluorescence employing a BV-2A filter (Nikon) and examined for their CTC staining patterns: (F) Pattern F sperm cell. (I) Pattern B sperm cell. (L) Pattern AR sperm cell.

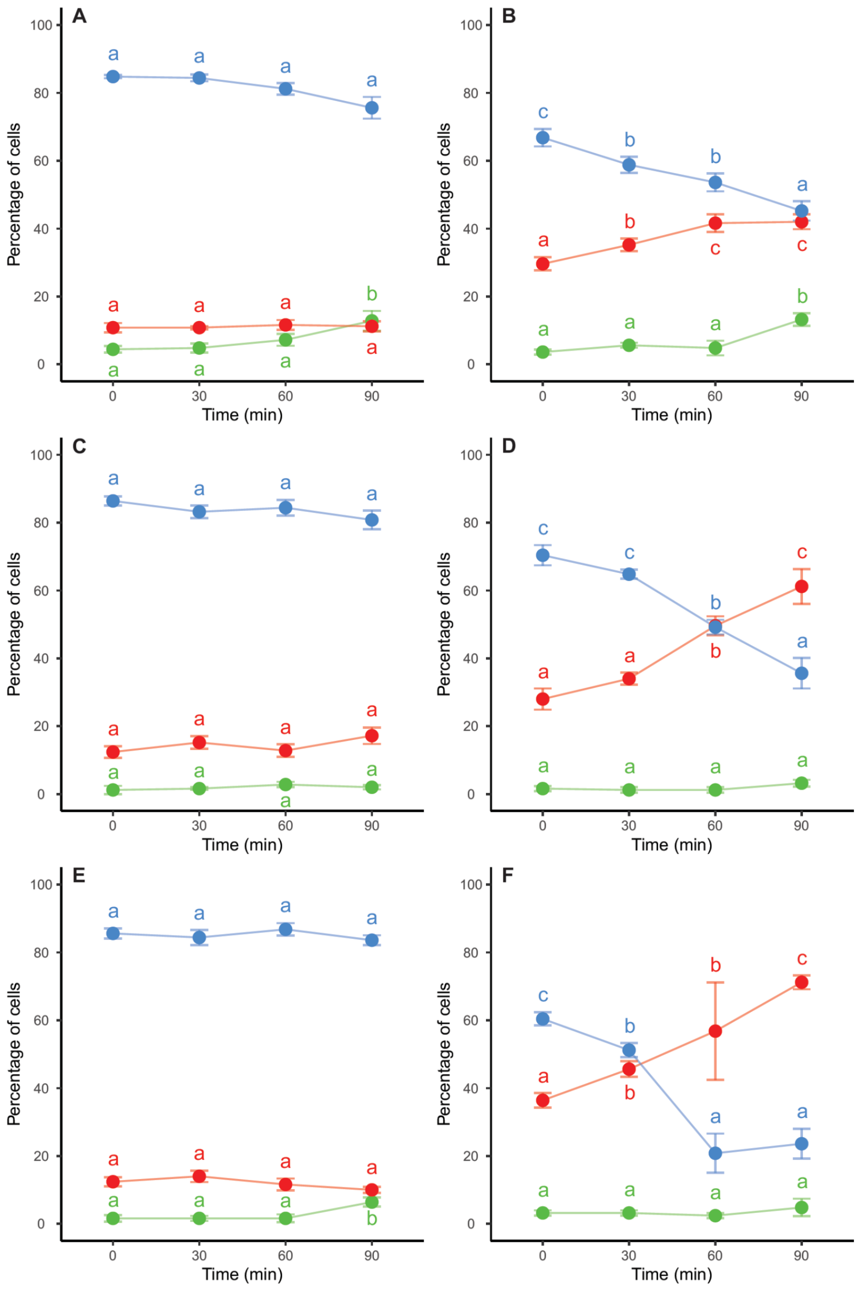

Figure S2. Changes in chlortetracycline staining patterns in (A, B) *M. musculus*, (C, D) *M. spretus* and (E, F) *M. spicilegus* incubated under noncapacitating conditions (A, C, E) or capacitating conditions (B, D, F). Data are means ± SE (n = 5 in *M. musculus*, n = 5 in *M. spretus* and n = 5 in *M. spicilegus*) of percentage of cells showing the corresponding pattern. Blue circles: F pattern (non-capacitated sperm). Red circles: B pattern (capacitated sperm). Green circles: AR pattern (spontaneous acrosome rection). Different letters indicate significant differences ($p < 0.05$) between times for the same variable in a Fisher *post-hoc* test.

Table S1. Effect of time of incubation ("Time") and incubation under noncapacitating or capacitating conditions ("Treatment") on the percentage of spermatozoa showing AR or F patterns after chlortetracycline (CTC) staining in *M. musculus*, *M. spretus* and *M. spicilegus*. Analyses for percentages of B pattern are shown in Table 1. Values *F* and *p* correspond to repeated measures ANOVA's. Individual was treated as random factor and incubation conditions and time as fixed factors. Results in bold show significant differences ($p < 0.05$) in statistic analyses.

**Dependent variable**	**Independent variable**	***Mus musculus***		***M. spretus***		***M. spicilegus***	
		F	***p***	***F***	***p***	***F***	***p***
AR pattern (%)	Treatment	0.183	0.672	0.032	0.860	0.439	0.512
	Time	**12.022**	**<0.001**	1.041	0.39	**3.431**	**0.029**
	Interaction	0.379	0.769	1.125	0.356	0.699	0.559
F pattern (%)	Treatment	**276.978**	**<0.001**	**225.360**	**<0.001**	**506.002**	**<0.001**
	Time	**18.512**	**<0.001**	**21.373**	**<0.001**	**23.209**	**<0.001**
	Interaction	**3.028**	**0.046**	**13.267**	**<0.001**	**23.660**	**<0.001**
